# Anterior dislocation of the hip associated with intertrochanteric fracture of the femur-Case presentation-

**Published:** 2013-09-25

**Authors:** R Radulescu, A Badila, I Japie, A Papuc, R Manolescu

**Affiliations:** * "Carol Davila" University of Medicine and Pharmacy, Bucharest; ** Department of Orthopedic Surgery, Bucharest University Hospital, Romania

**Keywords:** hip dislocation, intertrochanteric fracture, high energy trauma

## Abstract

Dislocations of the hip usually occur following high energy trauma, the coxo-femoral joint being inherently stable, and can be associated with acetabular fractures or fractures of the head, neck or shaft of femur. However, the combination between the anterior hip dislocation and the ipsilateral intertrochanteric fracture is extremely rare, the literature offering only scarce information.

We present the case of a patient, aged 44, victim of a trauma by precipitation from height (12m), diagnosed with left hip anterior dislocation and intertrochanteric fracture of the ipsilateral femur. An emergency surgical treatment was applied in less than 3 hours after trauma. The hip dislocation was reduced under general anesthesia and the intertrochanteric fracture was also reduced and internally fixed with a dynamic hip screw. Radiological and functional evaluation at 6 months after surgery, using the modified Merle D’Aubigne hip score was good.

The clinical outcome of such a case depends on the quick evaluation and treatment. Providing a stable reduction of the dislocation and a stable internal fixation of the fracture as soon as possible (within the first 6 hours) will allow an early physical rehabilitation and decrease the risk of complications.

## Introduction

The anatomic features of the coxo-femoral joint imply a high degree of stability. That is why dislocations of the hip usually occur following a high energy trauma, such as road traffic accidents, industrial accidents, sport injuries (e.g. soccer, rugby, and wrestling) or falls from a height [**1**], and it may be associated with acetabular fractures or fractures of the head, neck or shaft of femur. Posterior hip dislocation is approximately 9 times more frequent than the anterior type [**2,3**].

 The combination between the anterior hip dislocation and the ipsilateral intertrochanteric fracture, with the femoral head remaining intact, is extremely rare, only a few cases being reported in the literature.

 The aim of this paper is to present the case of a patient, aged 44, victim of a trauma by precipitation from height (12m), diagnosed with anterior left hip dislocation and intertrochanteric fracture of the ipsilateral femur.

## Clinical case

A 44-year-old male is admitted in the Emergency Room after he suffered a trauma by precipitation from height (12m). The patient is conscious, cooperative, hemodynamically stable, complaining of severe pain in the left hip, being unable to stand or bear weight on the left lower limb, which appears abducted and externally rotated, with no signs of any peripheral neurovascular lesions. In the groin area, an ovoid formation of hard consistency can be detected by palpation. Clinical and laboratory examinations exclude other coexisting abdominal, thoracic, neurological or musculoskeletal lesions.

**Fig. 1:**
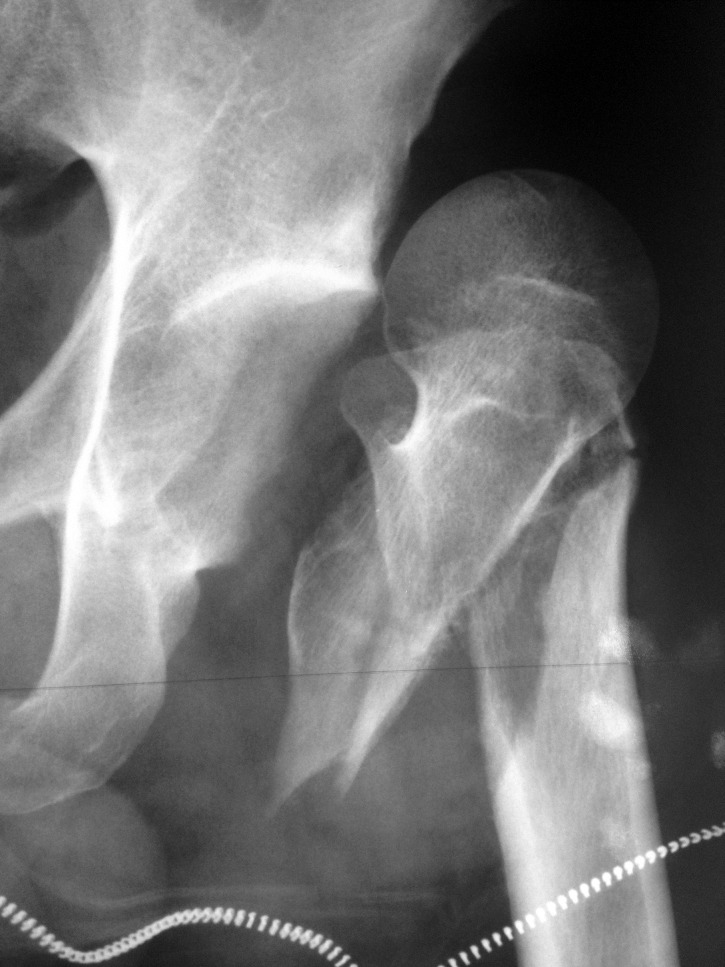
Antero-posterior X-Ray view of the left hip showing anterior displacement of the femoral head and intertrochanteric fracture

**Fig. 2:**
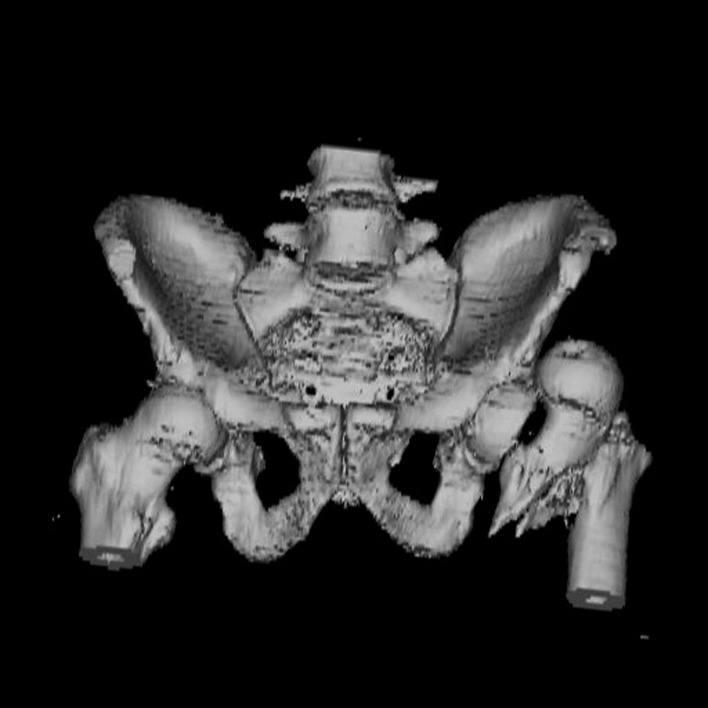
3D CT reconstruction of the pelvis showing anterior displacement of the left femoral head and intertrochanteric fracture. No other osteoarticular injuries were found

 An X-ray examination of the pelvis (**Fig. 1**) and a CT scan of the hip (**Fig. 2**) are performed and they confirm the diagnosis of type IB Epstein anterior hip dislocation associated with displaced intertrochanteric fracture of the femur.

 An emergency surgical treatment was applied in less than 3 hours since the trauma occurred. The hip dislocation was reduced under general anesthesia and the intertrochanteric fracture was also reduced and internally fixed with a dynamic hip screw (DHS).

 Under general anesthesia, a lateral approach of the hip was used, extended proximally and slightly anterior. After the superficial layers are incised, the femoral head is identified and reduced into the acetabulum by hand. Under fluoroscopic control, the intertrochanteric fracture was also reduced and internally fixed with DHS attached to a side plate with 5 screws. At the end of surgery, the stability of the hip is checked by performing a 90 degrees flexion of the thigh, internal-external rotation and abduction-adduction.

 Postoperatively, the patient is kept at rest in bed for 6 weeks and antithrombotic prophylaxis is initiated. Physical therapy is started immediately after surgery by predominantly isometric exercises for toning the muscles, at 3 weeks hip joint mobilization exercises and later (at 6 weeks after surgery) walking without weight bearing on the left lower limb is started. Progressive weight bearing of the injured limb is allowed at 3 months after surgery.

**Fig. 3:**
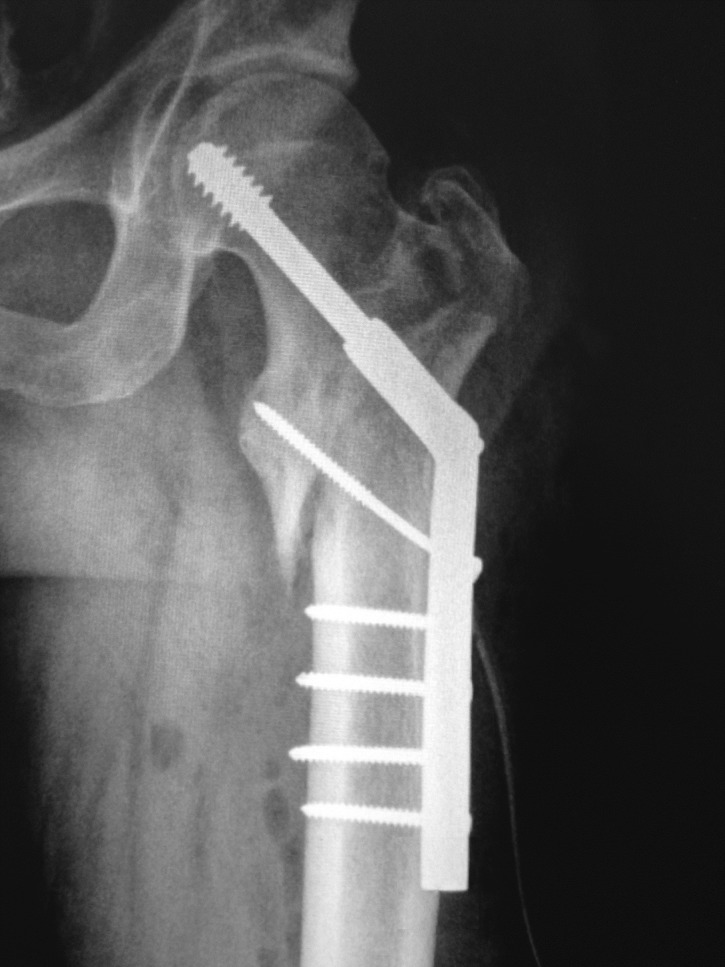
Postoperative antero-posterior X-ray view revealing fracture and dislocation reduction and fixation with dynamic hip screw

**Fig. 4:**
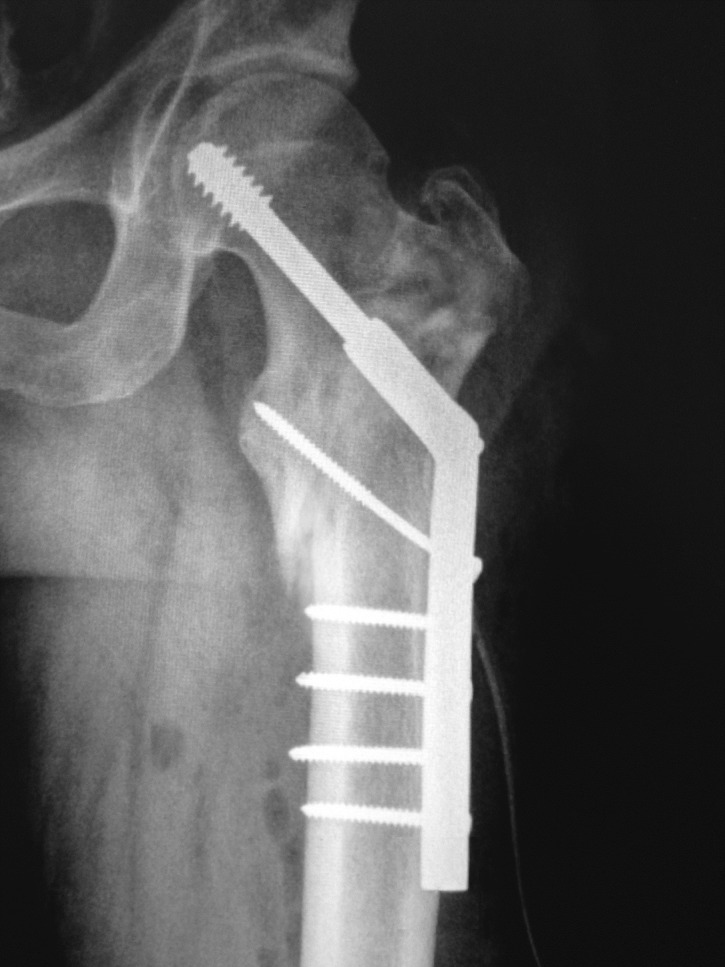
6 weeks postoperative antero-posterior X-ray view of the left hip

 Post-operative follow-up was assessed by radiological and functional criteria. X-rays examinations were performed immediately after surgery (**Fig. 3**), at 6 weeks (**Fig. 4**), at 6 months and at one year. 

 Functional evaluation was carried out at 6 months after surgery, according to Merle d'Aubigné score modified by Matta JM [**7**] (**Table 1**), which takes into account the presence of pain, ability to walk and joint range of motion. The measured functional score was 15 points out of a maximum of 18, signifying a good post-operative result.

**Table 1:** Merle d'Aubigné score modified by Matta JM

Points*	Pain	Walking	Range of Motion**
6	None	Normal	95-100%
5	Slight or intermittent	No cane, but slight limp	80-94%
4	After walking but resolves	Long distance with cane or crutch	70-79%
3	Moderately severe but patient is able to walk	Limited, even with support	60-69%
2	Severe, prevents walking	Very limited	50-59%
1	-	Unable to walk	< 50%
*Clinical grade: • 18 – Excellent • 15-17 – Good • 13, 14 – Fair • <13 – Poor. **The range of motion is expressed as the percentage of the value for the normal hip. This is calculated by obtaining a total of the ranges, in degrees, of flexion-extension, abduction, adduction, external rotation, and internal rotation for the injured hip and dividing it by the total for the normal hip.

## Discussions

The hip is a spheroidal type of joint with a good congruence between the femoral head and the acetabulum and reinforced by a thick articular capsule and strong ligaments. All these anatomical features make the hip joint very stable. That is why, hip dislocations usually occur following significant trauma. Hip dislocations can be posterior (most frequent) and anterior (10-15%). The anterior dislocations are described by the Epstein Classification [**4**]:

 • Type I - Superior dislocations

 - IA: no associated fractures

 - IB: associated fracture or impaction of the femoral head

 - IC: associated fracture of the acetabulum

 • Type II - Inferior dislocations

 - IIA: no associated fractures

 - IIB: associated fracture or impaction of the femoral head

 - IIC: associated fracture of the acetabulum

 Anterior dislocations usually result after a high energy trauma, which determines forced abduction and external rotation and of the hip. Depending on the position of the hip at the time of the impact, dislocations may be anterior-inferior (if the hip is in flexion) or anterior-superior (If the hip is in extension).

 The main peculiarity of the presented case is the association of an anterior-superior dislocation of the hip with ipsilateral intertrochanteric fracture. The latter can be explained by the developing of powerful forces that acted on the lateral aspect of the greater trochanter or by the impact of the greater trochanter against the iliac bone in a forced abduction and external rotation position of the hip.

 Another rare aspect of this case is the lack of acetabulum fractures. Although the forces that acted during the trauma were strong enough to lead to dislocation of the hip (a very strong articulation) and fracture of the femur (the strongest long bone in the body), they did not produce any bone lesion in the acetabulum. Frequently, hip dislocation is associated with acetabulum fractures. Forces acting on the femoral head of the femur put high pressure on the walls of the acetabulum, exceeding their strength, breaking them, thus creating new spaces for the dislocation.

 Finally, the absence of the associated lesions is also peculiar to this case. Generally speaking, due to high energy trauma forces involved in the process, abdominal and thoracic visceral injuries, neurological or other musculoskeletal lesions can frequently occur but overwhelmed by the dominant hip symptoms. Consequently, a careful general examination of the patient is mandatory in order accomplish a complete diagnosis.

 Hip dislocation is an orthopedic emergency that must be addressed to the hospital as soon as possible and its reduction must be accomplished as soon as the patient's condition allows anesthesia and surgery, in order to avoid further complications.

 The main complications that can occur following hip dislocation are represented by avascular necrosis of the femoral head, leading in time to osteoarthritis, heterotopic ossification around the joint and paralysis of the sciatic nerve.

The most feared late complication of hip dislocation is avascular necrosis of the femoral head. This complication is thought to be multifactorial; on one hand, during dislocation, the vascular network emerging from the trochanteric area is damaged together with the joint capsule and the round ligament artery together with the ligament. On the other hand, Duncan and Shim [**5**] demonstrated a functional disruption of cephalic circulation by a spasm of the large artery or of the cervical branches, with no organic lesion itself. If we take into consideration this mechanism, the early reduction of the dislocated hip decreases the risk of avascular necrosis. A delay of more than 6 hours increases the risk of avascular necrosis from 10 to 40% [**6**].

 In time, avascular necrosis of the femoral head leads to osteoarthritis of the hip. This is seen more frequently in posterior dislocations associated with fractures of the posterior wall of the acetabulum than in anterior dislocations, the main reason referring to the subchondral lesions determined by the impact of the femoral head with the acetabulum. Osteoarthritis incidence can be minimized by early anatomical reduction of the dislocation and the associated articular fractures, thus restoring articular congruence.

 Heterotopic ossification and palsy of the sciatic nerve were mostly observed in fractures associated with posterior dislocation of the hip.

## Conclusions

The clinical outcome of such a case depends on a rapid evaluation and treatment. Usually, these cases require multidisciplinary teams (orthopedics, E&A, intensive care, imaging, other medical and surgical specialties - general surgery, neurosurgery, thoracic surgery, cardiovascular surgery, internal medicine etc.) which can evaluate the patient’s status and vital functions, determine the exact local lesions, identify any associated injuries, ensure a good anesthesia during surgery and supportive intensive care measures. Providing a stable reduction of the dislocation and a firm internal fixation of the fracture as soon as possible (within the first 6 hours) will allow an early physical rehabilitation and decrease the risk of complications.
